# 4-[(2-Benzoyl-4-chlorophenyl)diazenyl]-3-methyl-1-phenyl-1*H*-pyrazol-5(4*H*)-one

**DOI:** 10.1107/S1600536809004243

**Published:** 2009-02-11

**Authors:** Zaker Bahreni, Hossein Rahmani, Seik Weng Ng

**Affiliations:** a Institute of Chemical Industries, Iranian Research Organization for Science and Technology, PO Box 15815-358, Tehran, Iran; b Department of Chemistry, University of Malaya, 50603 Kuala Lumpur, Malaysia

## Abstract

In the title compound, C_23_H_17_ClN_4_O_2_, the amino H atom forms an intra­molecular hydrogen bond to the exocyclic carbonyl O atom as well as to the O atom of the benzoyl group.

## Related literature

For the crystal structure of 1-phenyl-3-methyl-4-(4′-chloro­phenyazo)-pyrazol-5-one, whose amino H atom is intra­molecularly hydrogen-bonded to the carbonyl O atom, see: Golinski *et al.* (1983 ▶).
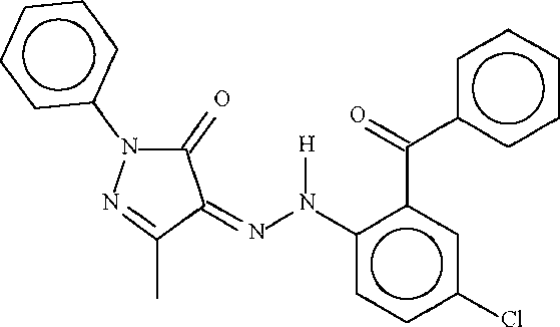


## Experimental

### 

#### Crystal data

C_23_H_17_ClN_4_O_2_
*M* *_r_* = 416.86
Monoclinic, 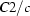

*a* = 25.800 (3) Å
*b* = 12.124 (1) Å
*c* = 13.966 (1) Å
β = 119.179 (1)°
*V* = 3813.9 (7) Å^3^
*Z* = 8
Mo *K*α radiation
μ = 0.23 mm^−1^
*T* = 120 (2) K
0.35 × 0.25 × 0.10 mm

#### Data collection

Bruker SMART APEX diffractometer
Absorption correction: multi-scan (*SADABS*; Sheldrick, 1996 ▶) *T*_min_ = 0.791, *T*_max_ = 0.862 (expected range = 0.897–0.977)
10753 measured reflections
4346 independent reflections
3309 reflections with *I* > 2σ(*I*)
*R*_int_ = 0.032

#### Refinement

*R*[*F* ^2^ > 2σ(*F* ^2^)] = 0.039
*wR*(*F* ^2^) = 0.105
*S* = 1.00
4346 reflections
276 parameters
1 restraint
H atoms treated by a mixture of independent and constrained refinement
Δρ_max_ = 0.30 e Å^−3^
Δρ_min_ = −0.30 e Å^−3^

### 

Data collection: *APEX2* (Bruker, 2008 ▶); cell refinement: *SAINT* (Bruker, 2008 ▶); data reduction: *SAINT*; program(s) used to solve structure: *SHELXS97* (Sheldrick, 2008 ▶); program(s) used to refine structure: *SHELXL97* (Sheldrick, 2008 ▶); molecular graphics: *X-SEED* (Barbour, 2001 ▶); software used to prepare material for publication: *publCIF* (Westrip, 2009 ▶).

## Supplementary Material

Crystal structure: contains datablocks global, I. DOI: 10.1107/S1600536809004243/tk2371sup1.cif

Structure factors: contains datablocks I. DOI: 10.1107/S1600536809004243/tk2371Isup2.hkl

Additional supplementary materials:  crystallographic information; 3D view; checkCIF report

## Figures and Tables

**Table 1:** Hydrogen-bond geometry (Å, °)

*D*—H⋯*A*	*D*—H	H⋯*A*	*D*⋯*A*	*D*—H⋯*A*
N4—H4⋯O1	0.89 (1)	2.06 (2)	2.755 (2)	135 (2)
N4—H4⋯O2	0.89 (1)	2.05 (2)	2.698 (2)	130 (2)

**Fig. 1.**
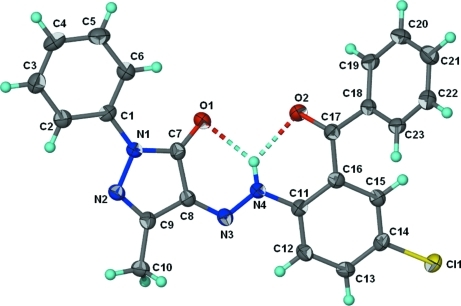
Thermal displacement plot (Barbour, 2001) of C23H17ClN4O2; probability levels are set at 70% and H-atoms are drawn as spheres of arbitrary radius.

## supplementary crystallographic information

### Experimental

2-Amino-5-chlorobenzophenone (2.32 g, 0.01 mol) was suspended in strong hydrochloric acid (20 ml, pH ca. 5) at 273 K. A solution of sodium nitrite (0.69 g in 15 ml water) was added. Following the diazotization, an aqueous solution of 3-methyl-1-phenyl-2-pyrazoline-5-one (1.75 g, 0.01 mol) was added. The compound that separated was collected and recrystallized from ethanol.

### Refinement

Carbon-bound H-atoms were placed in calculated positions (C—H 0.95 to 0.98 Å) and were included in the refinement in the riding model approximation, with *U*(H) set to 1.2 to 1.5*U*(C).

The amino H-atom was located in a difference Fourier map, and was refined with a distance restraint of N–H 0.85±0.01 Å; its temperature factors was freely refined.

### Crystal data

**Table:**

C_23_H_17_ClN_4_O_2_	*F*(000) = 1728
*M**_r_* = 416.86	*D*_x_ = 1.452 Mg m^−^^3^
Monoclinic, *C*2/*c*	Mo *K*α radiation, λ = 0.71073 Å
Hall symbol: -C 2yc	Cell parameters from 2643 reflections
*a* = 25.800 (3) Å	θ = 2.2–26.9°
*b* = 12.124 (1) Å	µ = 0.23 mm^−^^1^
*c* = 13.966 (1) Å	*T* = 120 K
β = 119.179 (1)°	Block, orange
*V* = 3813.9 (7) Å^3^	0.35 × 0.25 × 0.10 mm
*Z* = 8	

### Data collection

**Table:**

Bruker SMART APEX diffractometer	4346 independent reflections
Radiation source: fine-focus sealed tube	3309 reflections with *I* > 2σ(*I*)
graphite	*R*_int_ = 0.032
ω scans	θ_max_ = 27.5°, θ_min_ = 1.8°
Absorption correction: multi-scan (*SADABS*; Sheldrick, 1996)	*h* = −33→33
*T*_min_ = 0.791, *T*_max_ = 0.862	*k* = −15→12
10753 measured reflections	*l* = −18→17

### Refinement

**Table:**

Refinement on *F*^2^	Primary atom site location: structure-invariant direct methods
Least-squares matrix: full	Secondary atom site location: difference Fourier map
*R*[*F*^2^ > 2σ(*F*^2^)] = 0.039	Hydrogen site location: inferred from neighbouring sites
*wR*(*F*^2^) = 0.105	H atoms treated by a mixture of independent and constrained refinement
*S* = 1.00	*w* = 1/[σ^2^(*F*_o_^2^) + (0.05*P*)^2^ + 2.1067*P*] where *P* = (*F*_o_^2^ + 2*F*_c_^2^)/3
4346 reflections	(Δ/σ)_max_ = 0.001
276 parameters	Δρ_max_ = 0.30 e Å^−^^3^
1 restraint	Δρ_min_ = −0.30 e Å^−^^3^

### Fractional atomic coordinates and isotropic or equivalent isotropic displacement parameters (Å^2^)

**Table:**

	*x*	*y*	*z*	*U*_iso_*/*U*_eq_	
Cl1	0.176742 (18)	0.72052 (4)	0.10069 (4)	0.03104 (13)	
O1	0.51920 (5)	0.65437 (10)	0.64529 (9)	0.0270 (3)	
O2	0.42743 (6)	0.81878 (10)	0.49190 (10)	0.0301 (3)	
N1	0.56804 (6)	0.48796 (11)	0.72365 (11)	0.0209 (3)	
N2	0.55349 (6)	0.37433 (11)	0.70777 (11)	0.0208 (3)	
N3	0.42128 (6)	0.49431 (11)	0.50462 (11)	0.0200 (3)	
N4	0.40777 (6)	0.59936 (12)	0.47918 (11)	0.0204 (3)	
H4	0.4336 (7)	0.6518 (13)	0.5167 (15)	0.037 (6)*	
C1	0.62606 (7)	0.51899 (15)	0.80413 (13)	0.0214 (4)	
C2	0.66295 (7)	0.44153 (15)	0.88027 (13)	0.0239 (4)	
H2	0.6489	0.3691	0.8799	0.029*	
C3	0.72042 (8)	0.47068 (16)	0.95664 (14)	0.0279 (4)	
H3	0.7458	0.4175	1.0082	0.034*	
C4	0.74136 (8)	0.57598 (16)	0.95878 (15)	0.0307 (4)	
H4A	0.7810	0.5951	1.0108	0.037*	
C5	0.70394 (8)	0.65311 (16)	0.88431 (15)	0.0310 (4)	
H5	0.7179	0.7261	0.8866	0.037*	
C6	0.64650 (8)	0.62588 (15)	0.80647 (14)	0.0270 (4)	
H6	0.6213	0.6794	0.7552	0.032*	
C7	0.52131 (7)	0.55349 (14)	0.65290 (13)	0.0204 (3)	
C8	0.47428 (7)	0.47380 (14)	0.58702 (13)	0.0195 (3)	
C9	0.49904 (7)	0.36666 (14)	0.62833 (13)	0.0201 (3)	
C10	0.46871 (7)	0.25862 (14)	0.59029 (15)	0.0246 (4)	
H10A	0.4962	0.1992	0.6323	0.037*	
H10B	0.4559	0.2492	0.5122	0.037*	
H10C	0.4340	0.2562	0.6012	0.037*	
C11	0.35288 (7)	0.62787 (14)	0.38933 (13)	0.0198 (3)	
C12	0.30754 (7)	0.55002 (14)	0.34178 (13)	0.0226 (4)	
H12	0.3140	0.4770	0.3700	0.027*	
C13	0.25322 (7)	0.57835 (15)	0.25382 (14)	0.0241 (4)	
H13	0.2221	0.5255	0.2224	0.029*	
C14	0.24456 (7)	0.68414 (15)	0.21197 (13)	0.0221 (4)	
C15	0.28903 (7)	0.76156 (14)	0.25581 (13)	0.0213 (3)	
H15	0.2822	0.8336	0.2252	0.026*	
C16	0.34421 (7)	0.73499 (14)	0.34516 (13)	0.0200 (3)	
C17	0.39169 (7)	0.82058 (14)	0.39359 (13)	0.0210 (3)	
C18	0.39651 (7)	0.90830 (14)	0.32340 (13)	0.0205 (3)	
C19	0.42522 (7)	1.00593 (14)	0.37423 (14)	0.0251 (4)	
H19	0.4377	1.0172	0.4497	0.030*	
C20	0.43574 (8)	1.08644 (15)	0.31600 (15)	0.0298 (4)	
H20	0.4549	1.1531	0.3513	0.036*	
C21	0.41840 (8)	1.07047 (15)	0.20623 (15)	0.0299 (4)	
H21	0.4257	1.1259	0.1662	0.036*	
C22	0.39037 (8)	0.97341 (15)	0.15512 (14)	0.0273 (4)	
H22	0.3787	0.9623	0.0799	0.033*	
C23	0.37924 (7)	0.89228 (14)	0.21274 (13)	0.0230 (4)	
H23	0.3599	0.8259	0.1771	0.028*	

### Atomic displacement parameters (Å^2^)

**Table:**

	*U*^11^	*U*^22^	*U*^33^	*U*^12^	*U*^13^	*U*^23^
Cl1	0.0196 (2)	0.0284 (3)	0.0321 (2)	0.00060 (17)	0.00242 (17)	0.00243 (19)
O1	0.0295 (7)	0.0183 (7)	0.0261 (6)	−0.0014 (5)	0.0080 (5)	0.0012 (5)
O2	0.0339 (7)	0.0233 (7)	0.0217 (6)	−0.0045 (5)	0.0045 (5)	−0.0012 (5)
N1	0.0219 (7)	0.0173 (7)	0.0210 (7)	−0.0018 (6)	0.0086 (6)	0.0006 (5)
N2	0.0209 (7)	0.0177 (7)	0.0238 (7)	−0.0010 (6)	0.0109 (6)	0.0014 (6)
N3	0.0219 (7)	0.0200 (8)	0.0204 (7)	0.0008 (6)	0.0121 (6)	0.0028 (6)
N4	0.0210 (7)	0.0181 (8)	0.0195 (7)	−0.0015 (6)	0.0079 (6)	0.0013 (6)
C1	0.0192 (8)	0.0252 (9)	0.0193 (8)	−0.0022 (7)	0.0089 (7)	−0.0021 (7)
C2	0.0247 (9)	0.0248 (10)	0.0229 (8)	−0.0011 (7)	0.0121 (7)	−0.0003 (7)
C3	0.0263 (9)	0.0311 (10)	0.0233 (9)	0.0028 (8)	0.0097 (7)	0.0006 (8)
C4	0.0226 (9)	0.0373 (11)	0.0264 (9)	−0.0057 (8)	0.0073 (7)	−0.0078 (8)
C5	0.0309 (10)	0.0271 (10)	0.0325 (10)	−0.0085 (8)	0.0133 (8)	−0.0061 (8)
C6	0.0272 (9)	0.0239 (10)	0.0270 (9)	−0.0015 (7)	0.0109 (7)	0.0012 (7)
C7	0.0229 (8)	0.0209 (9)	0.0180 (8)	0.0010 (7)	0.0105 (7)	0.0024 (6)
C8	0.0209 (8)	0.0204 (9)	0.0184 (8)	−0.0010 (6)	0.0106 (7)	0.0017 (6)
C9	0.0193 (8)	0.0224 (9)	0.0205 (8)	0.0006 (7)	0.0111 (7)	0.0029 (7)
C10	0.0216 (8)	0.0198 (9)	0.0295 (9)	−0.0019 (7)	0.0102 (7)	−0.0006 (7)
C11	0.0184 (8)	0.0223 (9)	0.0185 (8)	0.0008 (6)	0.0090 (7)	−0.0007 (6)
C12	0.0245 (8)	0.0191 (9)	0.0251 (9)	−0.0001 (7)	0.0129 (7)	0.0036 (7)
C13	0.0211 (8)	0.0218 (9)	0.0276 (9)	−0.0047 (7)	0.0106 (7)	−0.0006 (7)
C14	0.0157 (8)	0.0261 (9)	0.0208 (8)	0.0021 (7)	0.0062 (6)	0.0000 (7)
C15	0.0214 (8)	0.0180 (9)	0.0233 (8)	0.0026 (6)	0.0100 (7)	0.0007 (7)
C16	0.0211 (8)	0.0193 (9)	0.0205 (8)	0.0003 (7)	0.0107 (7)	−0.0011 (6)
C17	0.0204 (8)	0.0178 (8)	0.0212 (8)	0.0024 (6)	0.0074 (7)	−0.0016 (7)
C18	0.0168 (7)	0.0180 (9)	0.0229 (8)	0.0016 (6)	0.0067 (6)	0.0008 (7)
C19	0.0231 (8)	0.0214 (9)	0.0248 (9)	−0.0009 (7)	0.0070 (7)	−0.0036 (7)
C20	0.0294 (9)	0.0188 (9)	0.0353 (10)	−0.0049 (7)	0.0110 (8)	−0.0018 (8)
C21	0.0300 (10)	0.0241 (10)	0.0348 (10)	−0.0013 (8)	0.0151 (8)	0.0053 (8)
C22	0.0266 (9)	0.0275 (10)	0.0247 (9)	0.0021 (7)	0.0099 (7)	0.0021 (7)
C23	0.0207 (8)	0.0192 (9)	0.0247 (9)	−0.0007 (7)	0.0076 (7)	−0.0019 (7)

### Geometric parameters (Å, °)

**Table:**

Cl1—C14	1.738 (2)	C10—H10A	0.9800
O1—C7	1.227 (2)	C10—H10B	0.9800
O2—C17	1.225 (2)	C10—H10C	0.9800
N1—C7	1.378 (2)	C11—C12	1.394 (2)
N1—C1	1.416 (2)	C11—C16	1.408 (2)
N1—N2	1.417 (2)	C12—C13	1.383 (2)
N2—C9	1.301 (2)	C12—H12	0.9500
N3—C8	1.312 (2)	C13—C14	1.382 (2)
N3—N4	1.323 (2)	C13—H13	0.9500
N4—C11	1.402 (2)	C14—C15	1.374 (2)
N4—H4	0.885 (9)	C15—C16	1.398 (2)
C1—C2	1.390 (2)	C15—H15	0.9500
C1—C6	1.393 (2)	C16—C17	1.492 (2)
C2—C3	1.385 (2)	C17—C18	1.492 (2)
C2—H2	0.9500	C18—C19	1.393 (2)
C3—C4	1.381 (3)	C18—C23	1.397 (2)
C3—H3	0.9500	C19—C20	1.380 (3)
C4—C5	1.382 (3)	C19—H19	0.9500
C4—H4A	0.9500	C20—C21	1.385 (3)
C5—C6	1.384 (3)	C20—H20	0.9500
C5—H5	0.9500	C21—C22	1.383 (3)
C6—H6	0.9500	C21—H21	0.9500
C7—C8	1.471 (2)	C22—C23	1.387 (2)
C8—C9	1.438 (2)	C22—H22	0.9500
C9—C10	1.485 (2)	C23—H23	0.9500
			
C7—N1—C1	129.3 (2)	H10B—C10—H10C	109.5
C7—N1—N2	112.1 (1)	C12—C11—N4	120.4 (2)
C1—N1—N2	118.7 (1)	C12—C11—C16	119.9 (2)
C9—N2—N1	107.2 (1)	N4—C11—C16	119.7 (1)
C8—N3—N4	116.3 (1)	C13—C12—C11	120.4 (2)
N3—N4—C11	119.8 (1)	C13—C12—H12	119.8
N3—N4—H4	121 (1)	C11—C12—H12	119.8
C11—N4—H4	120 (1)	C12—C13—C14	119.4 (2)
C2—C1—C6	120.0 (2)	C12—C13—H13	120.3
C2—C1—N1	119.5 (2)	C14—C13—H13	120.3
C6—C1—N1	120.5 (2)	C15—C14—C13	121.2 (2)
C3—C2—C1	119.6 (2)	C15—C14—Cl1	118.9 (1)
C3—C2—H2	120.2	C13—C14—Cl1	119.9 (1)
C1—C2—H2	120.2	C14—C15—C16	120.4 (2)
C4—C3—C2	120.9 (2)	C14—C15—H15	119.8
C4—C3—H3	119.6	C16—C15—H15	119.8
C2—C3—H3	119.6	C15—C16—C11	118.6 (2)
C3—C4—C5	119.2 (2)	C15—C16—C17	119.9 (2)
C3—C4—H4A	120.4	C11—C16—C17	121.5 (2)
C5—C4—H4A	120.4	O2—C17—C18	119.4 (2)
C4—C5—C6	121.2 (2)	O2—C17—C16	119.9 (2)
C4—C5—H5	119.4	C18—C17—C16	120.7 (1)
C6—C5—H5	119.4	C19—C18—C23	119.0 (2)
C5—C6—C1	119.2 (2)	C19—C18—C17	117.7 (2)
C5—C6—H6	120.4	C23—C18—C17	122.9 (2)
C1—C6—H6	120.4	C20—C19—C18	120.6 (2)
O1—C7—N1	128.6 (2)	C20—C19—H19	119.7
O1—C7—C8	127.8 (2)	C18—C19—H19	119.7
N1—C7—C8	103.7 (1)	C19—C20—C21	120.2 (2)
N3—C8—C9	126.3 (2)	C19—C20—H20	119.9
N3—C8—C7	127.8 (2)	C21—C20—H20	119.9
C9—C8—C7	105.8 (1)	C20—C21—C22	119.7 (2)
N2—C9—C8	111.2 (2)	C20—C21—H21	120.1
N2—C9—C10	122.0 (2)	C22—C21—H21	120.1
C8—C9—C10	126.8 (2)	C21—C22—C23	120.5 (2)
C9—C10—H10A	109.5	C21—C22—H22	119.7
C9—C10—H10B	109.5	C23—C22—H22	119.7
H10A—C10—H10B	109.5	C22—C23—C18	119.9 (2)
C9—C10—H10C	109.5	C22—C23—H23	120.0
H10A—C10—H10C	109.5	C18—C23—H23	120.0
			
C7—N1—N2—C9	0.5 (2)	N3—N4—C11—C12	14.3 (2)
C1—N1—N2—C9	−178.1 (1)	N3—N4—C11—C16	−164.0 (1)
C8—N3—N4—C11	177.4 (1)	N4—C11—C12—C13	179.4 (2)
C7—N1—C1—C2	168.3 (2)	C16—C11—C12—C13	−2.4 (2)
N2—N1—C1—C2	−13.4 (2)	C11—C12—C13—C14	1.2 (3)
C7—N1—C1—C6	−12.6 (3)	C12—C13—C14—C15	0.3 (3)
N2—N1—C1—C6	165.8 (2)	C12—C13—C14—Cl1	179.4 (1)
C6—C1—C2—C3	−1.4 (3)	C13—C14—C15—C16	−0.7 (3)
N1—C1—C2—C3	177.8 (2)	Cl1—C14—C15—C16	−179.8 (1)
C1—C2—C3—C4	0.6 (3)	C14—C15—C16—C11	−0.5 (2)
C2—C3—C4—C5	0.8 (3)	C14—C15—C16—C17	−178.6 (2)
C3—C4—C5—C6	−1.4 (3)	C12—C11—C16—C15	2.0 (2)
C4—C5—C6—C1	0.6 (3)	N4—C11—C16—C15	−179.8 (1)
C2—C1—C6—C5	0.8 (3)	C12—C11—C16—C17	−179.9 (2)
N1—C1—C6—C5	−178.4 (2)	N4—C11—C16—C17	−1.6 (2)
C1—N1—C7—O1	−1.6 (3)	C15—C16—C17—O2	148.1 (2)
N2—N1—C7—O1	180.0 (2)	C11—C16—C17—O2	−30.0 (2)
C1—N1—C7—C8	177.7 (2)	C15—C16—C17—C18	−32.3 (2)
N2—N1—C7—C8	−0.7 (2)	C11—C16—C17—C18	149.6 (2)
N4—N3—C8—C9	−179.0 (2)	O2—C17—C18—C19	−23.7 (2)
N4—N3—C8—C7	−1.8 (2)	C16—C17—C18—C19	156.7 (2)
O1—C7—C8—N3	2.3 (3)	O2—C17—C18—C23	149.5 (2)
N1—C7—C8—N3	−177.0 (2)	C16—C17—C18—C23	−30.1 (2)
O1—C7—C8—C9	180.0 (2)	C23—C18—C19—C20	0.9 (3)
N1—C7—C8—C9	0.6 (2)	C17—C18—C19—C20	174.4 (2)
N1—N2—C9—C8	−0.1 (2)	C18—C19—C20—C21	−0.7 (3)
N1—N2—C9—C10	−179.3 (1)	C19—C20—C21—C22	0.1 (3)
N3—C8—C9—N2	177.3 (2)	C20—C21—C22—C23	0.3 (3)
C7—C8—C9—N2	−0.4 (2)	C21—C22—C23—C18	−0.1 (3)
N3—C8—C9—C10	−3.5 (3)	C19—C18—C23—C22	−0.5 (2)
C7—C8—C9—C10	178.8 (2)	C17—C18—C23—C22	−173.6 (2)

### Hydrogen-bond geometry (Å, °)

**Table:**

*D*—H···*A*	*D*—H	H···*A*	*D*···*A*	*D*—H···*A*
N4—H4···O1	0.89 (1)	2.06 (2)	2.755 (2)	135 (2)
N4—H4···O2	0.89 (1)	2.05 (2)	2.698 (2)	130 (2)
